# Femoral Tunnel Geometry and Graft Inclination Angles in Anterior Cruciate Ligament Reconstruction Using a Flexible Reamer System

**DOI:** 10.3390/medicina59061031

**Published:** 2023-05-26

**Authors:** Dhong-Won Lee, Dong-Hwan Lee, Sung-Gyu Moon, Ji-Hee Kang, Young-Je Woo, Woo-Jong Kim

**Affiliations:** 1 Department of Orthopaedic Surgery, Konkuk University Medical Center, Konkuk University School of Medicine, Seoul 05029, Republic of Korea; 2 Department of Radiology, Konkuk University Medical Center, Konkuk University School of Medicine, Seoul 05029, Republic of Korea; 3 Department of Orthopaedic Surgery, Soonchunhyang University Hospital Cheonan, Cheonan 31538, Republic of Korea

**Keywords:** anterior cruciate ligament reconstruction, anteromedial portal, flexible reamer, femoral tunnel

## Abstract

*Background and Objectives*: The aim of this study is to investigate the femoral tunnel geometry (femoral tunsnel location, femoral graft bending angle, and femoral tunnel length) on three-dimensional (3D) computed tomography (CT) and graft inclination on magnetic resonance imaging (MRI) after anatomic anterior cruciate ligament (ACL) reconstruction using a flexible reamer system. *Materials and Methods*: A total of 60 patients who underwent anatomical ACL reconstruction (ACLR) using a flexible reamer system were retrospectively reviewed. One day after the ACLR procedure was performed, all patients underwent three-dimensional computed tomography (3D-CT) and magnetic resonance imaging (MRI). The femoral tunnel location, femoral graft bending angle, femoral tunnel length, and graft inclination were assessed. *Results*: In the 3D-CTs, the femoral tunnel was located at 29.7 ± 4.4% in the posterior to anterior (deep to shallow) direction and at 24.1 ± 5.9% in the proximal to distal (high to low) direction. The mean femoral graft bending angle was 113.9 ± 5.7°, and the mean femoral tunnel length was 35.2 ± 3.1 mm. Posterior wall breakage was observed in five patients (8.3%). In the MRIs, the mean coronal graft inclination was 69.2 ± 4.7°, and the mean sagittal graft inclination was 52.4 ± 4.6°. The results of this study demonstrated that a comparable femoral graft bending angle and longer femoral tunnel length were observed compared with the reported outcomes from previous studies that used the rigid reamer system. *Conclusions*: ACLR using a flexible reamer system allowed for an anatomic femoral tunnel location and a comparable graft inclination to that of the native ACL. In addition, it achieved a tolerable femoral graft bending angle and femoral tunnel length.

## 1. Introduction

Of the various surgical methods used for anatomical anterior cruciate ligament (ACL) reconstruction (ACLR), femoral drilling methods that are independent of the tibial tunnel have garnered attention [1,2,3]. The recent purpose of anatomical reconstruction is not only to achieve anatomical neo-ACL reconstruction but also to preserve the residual ACL where possible [4,5]. The transportal technique, the representative independent femoral drilling method, has potential risks, such as iatrogenic femoral cartilage injury, short femoral tunnel, posterior wall breakage, and peroneal nerve injury [6,7]. To overcome such potential risks, hyperflexion of the knee needs to be maintained during femoral drilling, in which the surgical field is blocked by hyperflexion [8,9]. Nonetheless, hyperflexion is needed because a guide pin and reamer are rigid [8].

A flexible reamer system was developed to overcome the limitation of the transportal technique that uses the existing rigid reamer system. Because the femoral drilling in this system is performed without more than 120° of knee hyperflexion, securing the surgical field, a relatively long femoral tunnel can be generated [10]. This can reduce the incidence of posterior wall breakage and avoid the risk of peroneal nerve injury. Previous cadaver studies reported that a significantly longer femoral tunnel could be achieved with a flexible guide pin than with a rigid guide pin [11,12]. A recently published systemic review reported that a clinical application of a flexible guide pin resulted in a longer and more anteverted femoral tunnel than a rigid guide pin [13]. In an attempt to overcome the inherent limitations of the transportal technique, the retrograde drilling method was introduced [14]. However, it is known that it has not yet been successful in reducing the incidence of acute graft bending [15,16]. As a result, the authors chose to use the transportal technique with a flexible reamer.

Because the postoperative outcomes of ACLR are affected by not only the graft length within the tunnel but also the femoral graft bending angle and graft inclination angle, merely securing a long femoral tunnel is not sufficient to obtain a desirable outcome. It is known that the femoral graft bending angle affects graft stress within the femoral tunnel, leading to tunnel widening or graft failure when an acute graft bending angle is formed [17,18,19]. Although it is unclear if the maintenance of inclination in the native ACL during ACLR would help obtain functionally beneficial clinical outcomes, it has been reported that a larger graft inclination is associated with graft laxity [20,21].

However, there has not been a comprehensive analytical study on femoral tunnel geometry and graft inclination after ACLR using a flexible reamer system. Therefore, the aim of the current study as a case series is to investigate the femoral tunnel geometry (femoral tunnel location, femoral graft bending angle, and femoral tunnel length) on three-dimensional (3D) computed tomography (CT) and graft inclination on magnetic resonance imaging (MRI) after anatomic ACLR using a flexible reamer system. It was hypothesized that ACLR using a flexible reamer system would result in an anatomical femoral tunnel location and an average femoral tunnel length of 35 mm or more while maintaining a tolerable graft bending angle. In addition, it was hypothesized that it would provide a relatively native graft inclination.

## 2. Materials and Methods

### 2.1. Patients

The medical records, 3D CT images, and MRI scans of 65 patients who underwent anatomical ACLR using a flexible reamer system at our institute from September 2021 to June 2022 were retrospectively reviewed. The inclusion criteria were primary ACLR; single-bundle reconstruction; the use of a soft-tissue graft; and availability to attend 3D CT and MRI evaluations. The exclusion criteria were selective bundle reconstruction, multiple-ligament injury, and revision ACLR. Among the 65 patients, 5 were excluded due to revision ACLR (*n* = 3), multiligament injury for which combined ligament reconstruction was performed (*n* = 1), and no follow-up MRI (*n* = 1). Finally, 60 patients were enrolled in this study. This retrospective investigation was conducted following approval from the Ethics Committee of Konkuk University Medical Center (KUMC 2023-01-001).

### 2.2. Surgical Techniques

All procedures were performed by a single experienced surgeon (DWL) using a flexible reamer system (VersiTomic, Stryker; Kalamazoo, MI, USA). In all patients, soft-tissue grafts (a hamstring autograft or hamstring autograft + tibialis allograft) were used, the diameters of which were 8–9 mm. The diameters of the femoral and tibial tunnels were equal to the diameter of the graft.

Three portals were created using a 30° arthroscope [22]. The anterolateral (AL) portal was established at the level of the patellar inferior pole as close as possible to the lateral border of the patellar tendon as a viewing portal. The anteromedial (AM) portal was established at the level of the patellar inferior pole as close as possible to the medial border of the patellar tendon as a viewing portal or working portal. This was in a slightly higher position than conventional to avoid the “sword fighting phenomenon.” The accessory anteromedial portal (AAM) was located inferior and medial to the AM portal, just above the anterior horn of the medial meniscus. Care was taken to avoid chondral damage of the medial femoral condyle. This allows the ACL femoral attachment to be viewed through the AM portal while instruments are inserted through this AAM portal.

**Figure 1:**
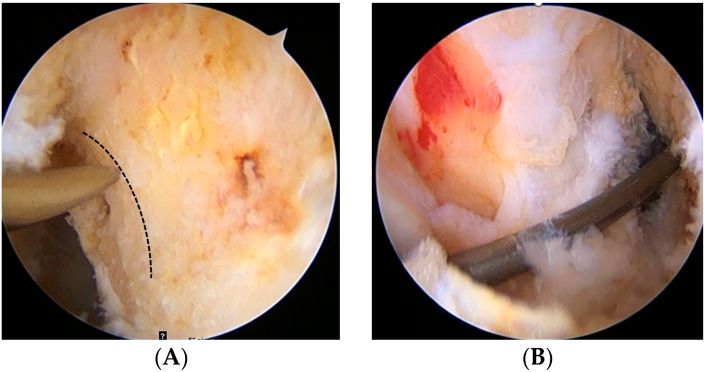

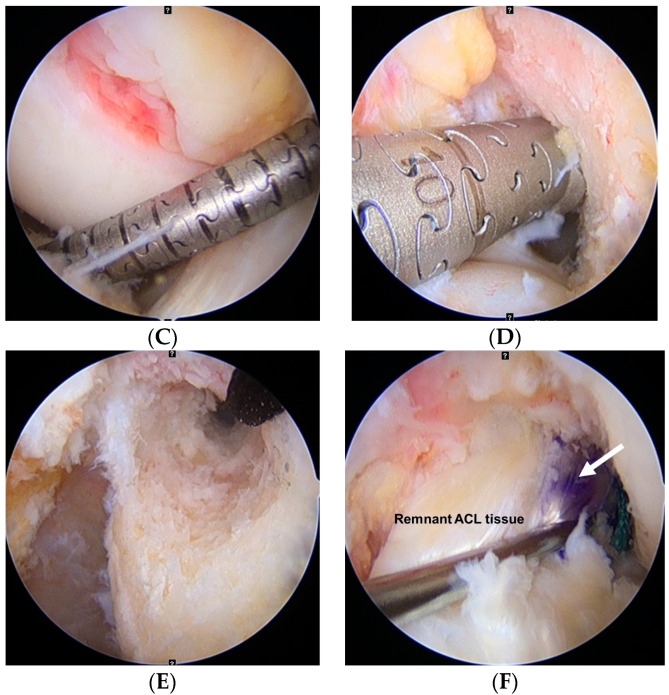
Anterior cruciate ligament (ACL) reconstruction of the left knee. (**A**) Anatomic center of the femoral tunnel is marked inferior to the lateral intercondylar ridge and just behind the bifurcate ridge (dotted line) using a microfracture awl through the accessory anteromedial (AM) portal, with the knee in 90° of flexion. (**B**) A flexible guide pin is inserted and drilled with the knee in 100° of flexion. (**C**) A flexible reamer of 4.5 mm diameter is drilled out through the far cortex to pass through a suspensory fixation device. (**D**) A flexible reamer of a diameter equal to that of the prepared graft is drilled. (**E**) The femoral tunnel is confirmed via the AM portal. (**F**) After tibial fixation, the reconstructed ACL graft containing the remnant tissue is evaluated.

The ACL femoral attachment was removed to identify the bony landmarks (lateral intercondylar ridge and bifurcate ridge) while preserving the remnant tissue substantially using electrocautery (ArthroCare, Smith & Nephew; Austin, TX, USA). While visualizing through the AL portal, the center of the femoral tunnel was marked inferior to the lateral intercondylar ridge and just behind the bifurcate ridge using a microfracture awl through the AAM portal, with the knee in 90° of flexion (Figure 1A). The tunnel center was determined using an arthroscopic bendable ruler (Smith & Nephew Endoscopy, Andover, MA, USA), ensuring that the posterior wall remained 2–3 mm. A 45° curved drill guide was introduced through the AAM portal to the center of the femoral tunnel with superolateral trajectory viewing through the AL portal. A flexible guide pin was inserted through the curved drill guide and drilled with the knee in 100° of flexion (Figure 1B). After the curved drill guide was removed, a flexible reamer of 4.5 mm diameter was drilled out through the far cortex to pass through a suspensory fixation device (Figure 1C). A flexible reamer of a diameter equal to that of the prepared graft was drilled at the same knee flexion angles (Figure 1D). The femoral tunnel was confirmed via the AM portal (Figure 1E). An ACL guide (Linvatec; Largo, FL, USA) was used to establish the tibial tunnel at an angle of 47.5°. The ACL guide tip was located just anteromedial to the center of the ACL bundles. A rigid reamer of a diameter equal to that of the prepared graft was drilled to protect the remnant ACL bundles. The prepared graft was passed from the tibial tunnel to the femoral tunnel. If the femoral tunnel length was 35 mm or longer, EndoButton (Smith & Nephew; Andover, MA, USA) with 15 mm tape was used for fixing. In contrast, if it was shorter than 35 mm, UltraButton (Smith & Nephew; Andover, MA, USA) was used for femoral fixation to maintain the 20 mm or longer graft length within the tunnel. The tibial side was dually fixed using a bioabsorbable interference screw (Matryx, ConMed Linvatec; Largo, FL, USA) and an additional cortical screw or staple with the knee in 20° of flexion. The diameter of the interference screw was equal to that of the tibial tunnel. Finally, the reconstructed ACL graft containing the original remnant ACL tissue was evaluated (Figure 1F).

### 2.3. Three-Dimensional CT Evaluation

One day after the ACLR procedure was performed, all patients underwent 3D CT scans (LightSpeed VCT XT, GE Medical Systems; Milwaukee, WI, USA). During the examination, the knee was extended as fully as possible.

**Figure 2:**
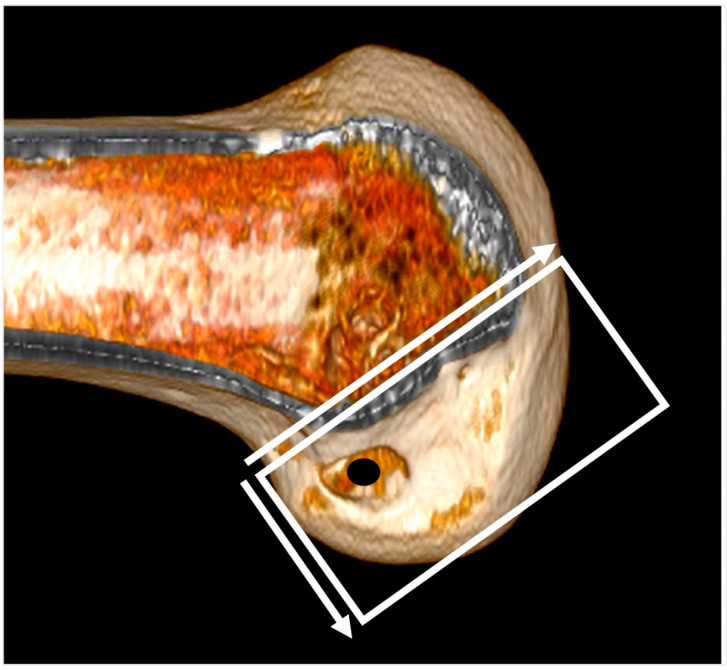
The femoral tunnel location is determined in the proximal to distal and anterior to posterior directions and presented as the percentage of the distance from the posterior edge of the lateral femoral condyle to the roof of the intercondylar notch.

To assess the femoral tunnel location, a true medial view of the lateral femoral condyle with the image of the medial femoral condyle erased at the center of the intercondylar notch was obtained. The quadrant methods used previously by Bernard et al. [23] and Forsythe et al. [24] facilitated the analysis of the femoral tunnel location (Figure 2). The tunnel location was determined in the proximal to distal (deep–shallow) and anterior to posterior (high–low) directions and presented as the percentage of the distance from the posterior edge of the lateral femoral condyle to the roof of the intercondylar notch. The measurements were performed on a picture archiving and communication system (PACS) workstation (Centricity RA 1000, GE Healthcare; Chicago, IL, USA).

**Figure 3:**
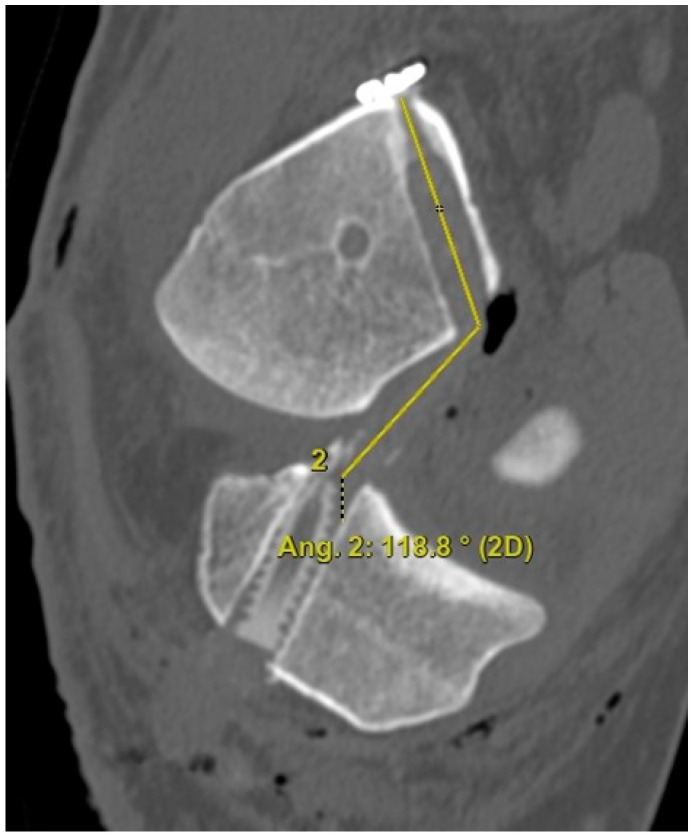
The femoral graft bending angle is defined as the angle formed by the longitudinal axis of the femoral tunnel and the line connecting the intra-articular aperture of the tibial tunnel and the intra-articular aperture of the femoral tunnel.

**Figure 4:**
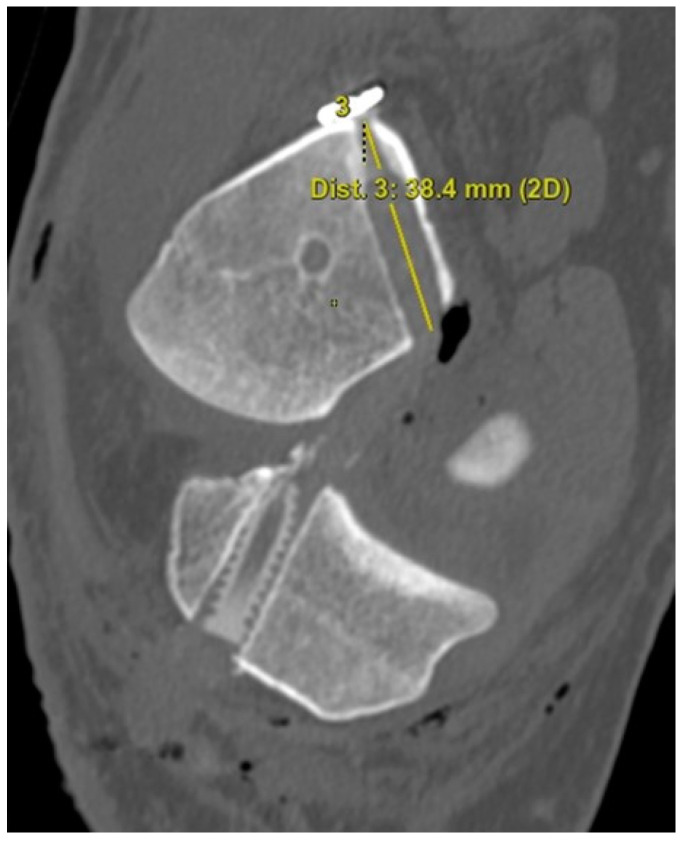
The femoral tunnel length is defined as the distance between the center of the extra-articular aperture of the femoral tunnel and the center of the intra-articular aperture of the femoral tunnel.

The 3D CT scans were imported into 3D software (AW Sever 3.2 PACS system, GE Healthcare; Chicago, IL, USA) for the assessments of the femoral graft bending angle and femoral tunnel length. The femoral graft bending angle was defined as the angle formed by the longitudinal axis of the femoral tunnel and the line connecting the intra-articular aperture of the tibial tunnel and the intra-articular aperture of the femoral tunnel (Figure 3). The femoral tunnel length was defined as the distance between the center of the extra-articular aperture of the femoral tunnel and the center of the intra-articular aperture of the femoral tunnel in a plane where the entire femoral tunnel could be viewed (Figure 4). Posterior wall breakage of the femoral tunnel was also checked.

### 2.4. MRI Evaluation

**Figure 5:**
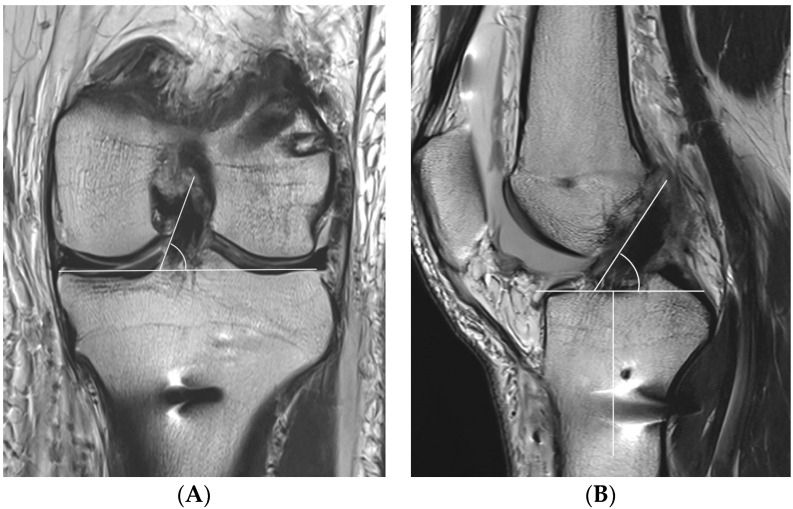
The coronal graft inclination (**A**) is defined as the angle between the medial margin of the anterior cruciate ligament (ACL) graft and a line parallel to the tibial plateau at the level of the middle of the medial collateral ligament. The sagittal graft inclination (**B**) is defined as the angle between the anterior margin of the ACL graft and a line perpendicular to the long axis of the tibia at the level of the Blumensaat line.

One day after ACLR, all patients underwent MRI with a 3.0-T system apparatus (Signa HD, GE Healthcare; Milwaukee, WI, USA) to evaluate for graft inclination. The graft inclination on the coronal plane was defined as the angle between the medial margin of the ACL graft through more than one slice and a line parallel to the tibial plateau at the level of the middle of the medial collateral ligament (Figure 5A). Because the entire ACL graft is unable to be visualized on one slice, if the medial margin of the ACL graft is marked and scrolled while watching the monitor, a line can be clearly drawn. The graft inclination on the sagittal plane was defined as the angle between the anterior margin of the ACL graft and a line perpendicular to the long axis of the tibia at the level of the Blumensaat line (Figure 5B).

### 2.5. Statistical Analysis

The statistical analysis was performed using SPSS software (IBM SPSS Statistics 21, IBM Corp.; Somers, NY, USA). Intrarater reliability was assessed using the intraclass correlation coefficient (ICC) with the following classifications: excellent (>0.9), good (0.75–0.9), moderate (0.5–0.75), and poor (<0.5). Interrater reliability between the examiners was assessed using kappa (k) values and classified as excellent (0.81–1.00), substantial (0.61–0.80), moderate (0.41–0.60), fair (0.21–0.40), or slight (0–0.20) [25]. All radiological measurements were independently performed by one experienced radiologist and one experienced orthopedic surgeon (SGM and DWL). Each investigator evaluated all images two times in intervals of 6 weeks and was blinded to the other investigator’s evaluation. The average data of two measures were used for the analysis.

## 3. Results

The basic demographics of enrolled patients are summarized in Table 1. The kappa values for intraobserver and interobserver agreements for the measurements were all >0.81 (Table 2).

Regarding the femoral tunnel location, it was located at 29.7 ± 4.4% in the posterior to anterior (deep to shallow) direction and at 24.1 ± 5.9% in the proximal to distal (high to low) direction. The mean femoral graft bending angle was 113.9 ± 5.7°, and the mean femoral tunnel length was 35.2 ± 3.1 mm (Table 3). There were no cases of a femoral tunnel length <30 mm because the guide pin was redirected for those <30 mm while making the knee joint flexion to approximately 110°. Posterior wall breakage was observed in five patients (8.3%). In the MRIs, the mean coronal graft inclination was 69.2 ± 4.7°, and the mean sagittal graft inclination was 52.4 ± 4.6° (Table 3). The mean posterior tibial slope 241 was 7.9 ± 3.5, and there was no posterior tibial slope of more than 12°.

There were no cases of iatrogenic chondral damage of the medial femoral condyle during the procedure. The mean operative time was 72.4 ± 12.1 min.

The results of this study were compared with those of previous studies in which an anatomical single-bundle ACLR using a flexible reamer system or rigid reamer system was performed (Table 4).

## 4. Discussion

The most substantial findings of the current study were that the flexible reamer system allowed for an anatomic femoral tunnel location and a comparable graft inclination to those of the native ACL. In addition, our results demonstrate that a comparable femoral graft bending angle and longer femoral tunnel length were observed compared with the reported outcomes from previous studies that used the rigid reamer system (Table 4).

Parkar et al. [29] published a systematic review of normal ACL center locations. In this systematic review, 218 knees demonstrated that the weighted 5th and 95th percentiles for the deep–shallow direction were 24% and 37%, respectively, and they were 28% and 43%, respectively, for the high–low direction. Piefer et al. [30] conducted a systematic review of the ACL femoral footprint anatomy. In their systematic review, the center of AM bundle ranged from 15% to 26.4% for the deep–shallow direction and 14.2% to 25.3% for the high–low direction. The center of the ACL footprint ranged from 23.5% to 43.1% for the deep–shallow direction and 27.5% to 44.25% for the high–low direction. In the present study, the center of the femoral tunnel was 28.8 ± 4.7% for the deep–shallow direction and 24.1 ± 5.9% for the high–low direction, and this femoral tunnel location was slightly closer to the AM bundle than the anatomical center of the ACL footprint. This indicates that the tunnel was well generated at the intended position because we targeted not the center between the AM bundle and the PL bundle but rather the region just posterior to the bifurcate ridge when marking the center of the femoral tunnel. To create a relatively consistent position of the femoral tunnel, we determined the tunnel center location using an arthroscopic bendable ruler (Smith & Nephew Endoscopy, Andover, MA, USA), ensuring that the posterior wall remained 2–3 mm.

In terms of graft inclination, Jamsher et al. [31] applied and compared four drilling techniques (the transportal technique using a flexible reamer system, the transportal technique using a rigid reamer system, outside–in retrograde drilling technique, and the transtibial drilling technique) for ACLR and demonstrated that the transportal technique using a flexible reamer system and outside–in retrograde drilling technique had outcomes more similar to those of sagittal and coronal inclinations than the transportal technique using a rigid reamer system and transtibial technique. In the study, the inclinations of the native ACL in the sagittal plane and coronal plane were 49.3 ± 4.2° and 73.6 ± 3.4°, respectively, and the graft inclination in ACLR with the transportal technique using a flexible reamer system in the sagittal plane and coronal plane were 49.9 ± 5.0° and 69.3 ± 4.5°, respectively. Other studies also reported that the sagittal inclination of the native ACL was 51–52° [20,32]. Snoj et al. [21] reported 52.2 ± 4.4° for the sagittal inclination of native ACL and 65.2 ± 6.6° for coronal inclination. Thus, the sagittal and coronal inclinations in our study were as close as within a 5° difference as those from inclinations of the native ACL reported by previous studies. Because a femoral offset guide was not used when applying the flexible reamer system, we were able to easily adjust the vertical position of the femur tunnel, which seemed to affect the outcome positively [31]. Although the effect of graft inclination on the clinical outcomes of ACLR has yet to be revealed, some report that it could have effects as considerable as the femoral tunnel location. Snoj et al. [21] demonstrated that there were significant correlations between anterior tibial translation and the sagittal (*p* = 0.01) and coronal (*p* < 0.01) inclinations. Hagiwara et al. [20] reported that the lateral sagittal inclination ranged from 45.8° to 65.4° after ACLR and that a larger graft inclination was significantly correlated with graft laxity.

Regarding the femoral graft bending angle, there are contradictory reports between the rigid reamer system and the flexible reamer system. Seo et al. [27] reported that the mean angles with the application of a flexible reamer system and a rigid reamer system for single-bundle ACLR were 109.8° and 118.1°, respectively, demonstrating a more acute outcome for the flexible reamer system. In contrast, Kim et al. [33] applied a flexible reamer system for double-bundle ACLR, resulting in 115.5° for a mean graft bending angle of AM bundle, which is less acute than that when a rigid reamer system was applied (108.4°). When applying a flexible reamer, Yoon et al. [26] made a tunnel in the lateral femoral condyle in a less perpendicular trajectory using a standard AM portal rather than an accessory AM portal. In our study, the mean graft bending angle was 113.9°, which is less acute than the mean angle (108.4°) reported by Yoon et al. [26]. Although we used an accessory AM portal, we first performed 4.5 mm reaming for a suspensory fixation system to generate a less acute graft bending angle and then tried reaming in the lateral femoral condyle in a less perpendicular trajectory while pushing back a thick reamer suitable for the graft size as far as possible. A sharp graft bending angle at the edge of the femoral tunnel entrance would load much stress on the graft, which could cause graft immaturation or failure, so it should be carefully applied in a flexible reamer system [19,34].

The mean femoral tunnel length (35.2 ± 3.1 mm) of the present study was longer than that of the rigid reamer system and similar to that of previous studies that used the flexible reamer system (Table 4). An advantage of the flexible reamer is that a desirable femoral tunnel length can be secured even without having knee flexion of 120° or greater [11,12]. Hence, if it is possible to generate a 30 mm or longer femoral tunnel, it should be better not to make a tunnel acute graft bending by hyperflexion for elongation of the tunnel. In the current study, a fixed loop suspensory device was used for 35 mm or longer femoral tunnels, whereas an adjustable loop suspensory device was utilized for those shorter than 35 mm to maintain a 20 mm or longer graft within the tunnel. It is known that there should be no clinical difference between them [35]. In comparison with the existing rigid reamer system, the application of the flexible reamer system not only secured a longer femoral tunnel length but also had fewer incidences of posterior wall breakage, which should be considered that it had overcome the weaknesses of the existing transportal technique. Some authors showed that there were only two cases of posterior 334 wall breakage-one in the flexible reamer group (*n* = 25) and one in the rigid reamer group (*n* = 25) [36]. However, their mean femoral tunnel position (deep to shallow) was 32.9% for the flexible reamer group and 32.8% for the rigid reamer group. Our femoral tunnel position was deeper since it was slightly closer to the AM bundle. In order to make a femoral tunnel close to the AM bundle, the risk of breakage is inevitably increased because it is close to the poster wall. Care should be taken to prevent poster wall breakage from occurring, even if the possibility of poster wall breakage is lower than that of the rigid reamer.

By analyzing 3D CT and MRI performed in previous studies, the present study investigated not only femoral tunnel geometry but also graft inclination. To date, there have been no reports of the long-term clinical effects of flexible reamers. Since this study generated tunnels using anatomic femoral footprints with the application of the flexible reamer system and was able to reproduce graft inclination close to native ACL, it should be considered significant, allowing the expectation of desirable long-term clinical outcomes.

This study is a comprehensive evaluation of 3D CT and MRI after ACLR in a relatively homogeneous group of 60 patients at a single institution. Based on the study results, when using a flexible reamer, it is possible to create an anatomically consistent femoral tunnel within a short surgery time, and because the graft bending angle is not acute, we believe that satisfactory radiological examination results, such as CT and MRI, can be obtained once one becomes familiar with this technique.

The current study had some limitations. First, this study was retrospective in design, and there was no control group. Second, since few studies have investigated the femoral tunnel geometry of ACLR, studies comparable to the radiological results of this study and their results have been insufficient. The reason for analyzing previous studies in the results is solely to supplement the data analysis of previous research, which may be insufficient in a case series. Third, this study did not assess the heterogeneity of the patient’s characteristics and the risk of bias in the included papers. Fourth, no clinical outcomes were included because this study analyzed 3D CT and MRI data right after surgery. Therefore, even a satisfactory radiological outcome should not be predicated on desirable clinical outcomes. Further research is needed on this. Fifth, while the femoral graft bending angle can be changed depending on the knee motion, this study evaluated the patients only in static states by CT, so it has a limitation in evaluating in vivo clinical significance. Finally, at least 1 year of postoperational MRI follow-up is required to evaluate the effect of the femoral geometry and graft inclination on graft maturation.

## 5. Conclusions

ACLR using a flexible reamer system allowed for an anatomic femoral tunnel location and a comparable graft inclination to that of the native ACL. In addition, it achieved a tolerable femoral graft bending angle and femoral tunnel length.

## Figures and Tables

**Table 1:** Demographic data.

	*N* = 60
Age, y	28.4 ± 9.9
Sex, *n*, Male/Female	37/23
Body Mass Index, kg/m^2^	23.2 ± 5.3
Side, *n*, Right/Left	29/31

**Table 2:** Reliability of each radiological measurement.

	Tunnel Location		FGBA	Tunnel Length	Graft Inclination	
	Posterior to Anterior	Proximal to Distal			Coronal	Sagittal
Examiner 1	0.91	0.89	0.89	0.97	0.91	0.94
Examiner 2	0.89	0.87	0.86	0.94	0.89	0.90
Interexaminer	0.86	0.84	0.85	0.96	0.89	0.91

FGBA, femoral graft bending angle.

**Table 3:** Femoral tunnel geometry and graft inclination.

	*N* = 60
Femoral tunnel location, %	
Proximal to distal (deep–shallow)	28.8 ± 4.7
Anterior to posterior (high–low)	24.1 ± 5.9
Femoral graft bending angle, °	113.9 ± 5.7
Femoral tunnel length, mm	35.2 ± 3.1
Graft inclination, °	
Coronal inclination	69.2 ± 4.7
Sagittal inclination	52.4 ± 4.6

**Table 4:** Current findings and previously reported femoral tunnel geometry in anatomical single-bundle anterior cruciate ligament reconstruction.

Study	Cases, n	Technique	Femoral Tunnel Location, %		Femoral Graft Bending Angle, °	Femoral Tunnel Length, mm	Posterior Wall Breakage, *n* (%)
			Deep–Shallow	High–Low			
Current study	60	TP (3 portals)/FR	28.8 ± 4.7	24.1 ± 5.9	113.9 ± 5.7	35.2 ± 3.1	5 (8.3)
Yoon et al. [26]	30	TP (2 portals)/FR	29.6 ± 5.5	20.1 ± 6.7	108.4 ± 6.9	32.8 ± 4.5	2 (6.6)
Seo et al. [27]	14	TP (3 portals)/FR	28.0 ± 6.3	23.7 ± 5.8	109.8 ± 9.4	36.7 ± 2.9	N/A
	14	TP (3 portals)/RR	32.1 ± 4.3	21.5 ± 5.2	118.1 ± 7.2	32.9 ± 9.0	
Park et al. [28]	21	TP (3 portals)/RR	26.7 ± 4.5	32.1 ± 8.0	107.9 ± 10.0	29.6 ± 3.9	7 (33.3)
	30	OI/RR	31.3 ± 6.0	27.6 ± 10.1	101.3 ± 8.2	33.0 ± 3.5	1 (3.3)

TP, transportal; FR, flexible reamer; RR, rigid reamer; OI, outside–in; N/A, Not Available.

## Data Availability

The data that support the findings of this study are available on request from the corresponding author.
